# Aseptic Technology for Cryoprotectant-Free Vitrification of Human Spermatozoa by Direct Dropping into Clean Liquid Air: Apoptosis, Necrosis, Motility, and Viability

**DOI:** 10.1155/2020/2934315

**Published:** 2020-01-24

**Authors:** Mengying Wang, Evgenia Isachenko, Plamen Todorov, Gohar Rahimi, Peter Mallmann, Igor I. Katkov, Vladimir Isachenko

**Affiliations:** ^1^Research Group for Reproductive Medicine and IVF-Laboratory, Department of Obstetrics and Genecology, Cologne University, Kerpener Str. 34, 50931 Cologne, Germany; ^2^Institute of Biology and Immunology of Reproduction, Tzarigradsko Shosse 73, 1113 Sofia, Bulgaria; ^3^Stem Cell Center, University of California at San Diego, La Jolla, CA 92037, USA

## Abstract

This study aimed to compare the quality of human spermatozoa vitrified by direct plunging into liquid nitrogen vs. liquid air. Spermatozoa were divided into three groups: fresh spermatozoa (Group F) were used as a control. Spermatozoa suspension (20 *μ*l) was vitrified in open granules by direct dropping into liquid nitrogen (Group LN) or clean liquid air (Group LA). After warming at 37°C, the progressive motility rate of Group F was reduced from 65.9 ± 2.5% to 34.0 ± 1.9% (Group LN) and 38.1 ± 2.3% (Group LA), respectively (P_1-2,3_ < 0.05). The reductions in viability were 65.6 ± 2.2%, 29.0 ± 1.8%, and 36.6 ± 2.6% for Groups F, LN, and LA, respectively (P_1-2,3_ < 0.05). Comparing spermatozoa vitrified in liquid nitrogen vs. liquid air, no significant differences were detected in motility (34.0 ± 1.9% vs. 38.1 ± 2.3%), viability (29.0 ± 1.8% vs. 36.6 ± 2.6%), early apoptosis (13.8 ± 1.5% vs. 14.3 ± 1.8%), late apoptosis (45.5 ± 1.8% vs. 43.7 ± 2.2%), and necrosis (19.5 ± 2.0% vs. 15.0 ± 1.8%; *p* > 0.01 for all respective differences). There was a statistical tendency for increasing rates of “progressive motility” and “viability” and decreasing rates of “apoptosis” and “necrosis” when comparing spermatozoa vitrified in liquid air vs. liquid nitrogen. It is concluded that cryoprotectant-free vitrification by the direct dropping of human spermatozoa in a clean cooling agent (liquid air) is a good alternative to the use of nonsterile liquid nitrogen and can be used to cool cells while minimising the risk of microbial contamination.

## 1. Introduction

Cryopreservation of spermatozoa is an important technology of reproductive medicine [1, 2]. Since data on the cryopreservation of human spermatozoa in the presence of cryoprotectants were first published in the late 1950s [3], several cryopreservation methods have been introduced, including conventional freezing and vitrification (cryopreservation by direct plunging into liquid nitrogen) techniques [4–6].

Conventional cryopreservation methods presuppose the use of cryoprotective agents (permeable cryoprotectants), which can be toxic [7]. Negative impacts of conventional cryopreservation on spermatozoa functions might also include intracellular ice crystal formation, cellular dehydration, osmotic injury, cytoplasm damage, functional destabilisation, and mutagenesis [8–11]. This method can lead to the alteration of membrane permeability [12–14]. To avoid these detrimental effects, cryoprotectant-free vitrification technology has been developed [13, 15–18].

It was noted the high effectiveness of technology for cryoprotectant-free vitrification by direct dropping of human spermatozoa suspension into liquid nitrogen [6, 19–21].

However, cryopreservation of nonisolated biological objects (with direct contact with liquid nitrogen) would take potential risks of contamination by viruses, bacteria, fungi, and spores [21–26].

To avoid the risk of microbial contamination, for cooling of cells, clean liquid air was used (bench-top device CLAir, FertileSafe, Nes Ziona, Israel) [27]. This bench-top device produces clean liquid air (CLAir, FertileSafe, Nes Ziona, Israel) having a temperature (−195.7°C) similar to temperature of liquid nitrogen. In experiments, Arav et al. [27] reported the use of device for the vitrification of mice embryos and human oocytes. The results showed that the devices are safe and can be easily adopted in every assisted reproduction treatment laboratory to eliminate potential contamination of cells by direct contact of these cells with liquid agent.

The vitrification of human pronuclear oocytes through direct contact with a sterile cooling agent (liquid air) has been recently reported [28]. But, there is limited data about the vitrification of human spermatozoa through direct contact with clean liquid air.

This study aimed to compare the motility, viability, and rates of apoptosis and necrosis of human spermatozoa vitrified in open granules, without permeable cryoprotectants, comparing direct plunging of the spermatozoa droplets into liquid nitrogen vs. liquid air.

## 2. Materials and Methods

All chemicals used in this study were purchased from Sigma (St. Louis, MO, USA), unless otherwise indicated. Our experiments were performed under a protocol approved by the University Ethics Board.

### 2.1. Selection and Preparation of Spermatozoa

Ejaculated semen samples were obtained from 15 healthy fertile men by masturbation after 48 h of sexual abstinence, who gave consent, and according to a protocol approved by the Ethics Boards of University Cologne.

Fresh semen samples were transported from the clinic to the laboratory within 20 min at 37°C. After liquefaction, a semen analysis was performed to evaluate the spermatozoa parameters, according to published guidelines of the World Health Organization [29].

A density-gradient medium (Gynemed GmbH, Lensahn, Germany) was prepared in a test tube by layering 1 ml of 45% (v/v) density-gradient medium over 1 ml of 90% (v/v) density-gradient medium. Then, 1 ml of semen was placed above the density-gradient media and centrifuged at 300–400 g for 15–30 min. Supernatant was removed from the spermatozoa pellet, and then spermatozoa pellet was resuspended in 5 ml of a supplemented medium by gentle pipetting and centrifuged at 200 g for 4 to10 min. Finally, the pellet in a supplemented medium was resuspended by gentle pipetting and concentration and motility of spermatozoa can be determined. Human tubal fluid (HTF) [30] with 1% Dextran Substitutive Supplement (DSS, Irvine Sci., Irvine, USA) was the basic medium for spermatozoa preparation.

Only normozoospermia samples were included in this study.

### 2.2. Experimental Design

**Figure 1:**
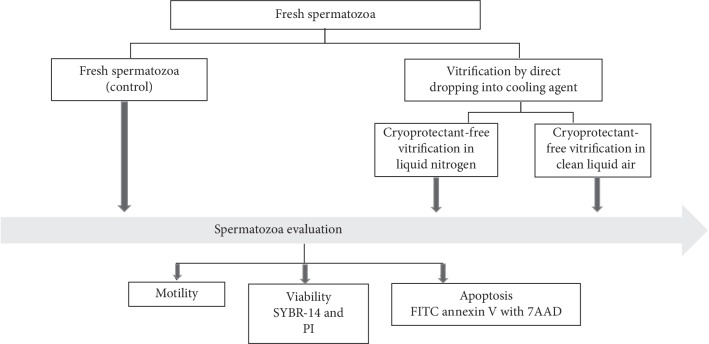
Experimental design. SYBR-14, membrane-permeant, nonfluorescent compound; PI, propidium iodide; FITC, fluorescein isothiocyanate; Annexin V, cellular protein; 7-AAD, 7-amino-actinomycin D.

The study design is presented in Figure 1. Spermatozoa were divided into three groups, one control and two treatment groups: cryopreserved in open granules by direct dropping into cooling agent (liquid nitrogen vs. liquid air). The quality of fresh spermatozoa, as well as spermatozoa after respective cryopreservation (vitrification and warming), was evaluated as described below.

### 2.3. Vitrification and Warming

CLAir (FertileSafe Ltd, Nes Ziona, Israel) is a bench-top device for the production of sterile liquid air made of two stainless steel containers, one inside the other, having a gap between them that is filled with commercial liquid nitrogen. The liquid air is produced inside the cooled, inner stainless steel container, which collects filtered room air (equipped with a 0.22 *μ*m filter) and liquefies it. The CLAir device produces 250 mL of clean liquid air every 10 min when operated according to the instruction manual. The liquid air is collected into a specially designed sterile Styrofoam cup, which can be used for cryopreservation with open carrier systems. Liquid air has the same temperature as liquid nitrogen (−195.7°C).

For the preparation of the vitrification solution, the 0.5 M stock of sucrose (MP Biomedicals, Illkirch, France) was dissolved into bidistillate water (Berlin-Chemie, Berlin, Germany) followed by filtration through a 0.22 *µ*m filter (Millipore, Darmstadt, Germany). The vitrification solution was prepared using extempore as follows: 0.5 M sucrose was diluted 1 : 1 with HTM medium supplemented with 1% DSS, to achieve the 0.25 M sucrose end concentration [4, 13].

**Figure 2:**
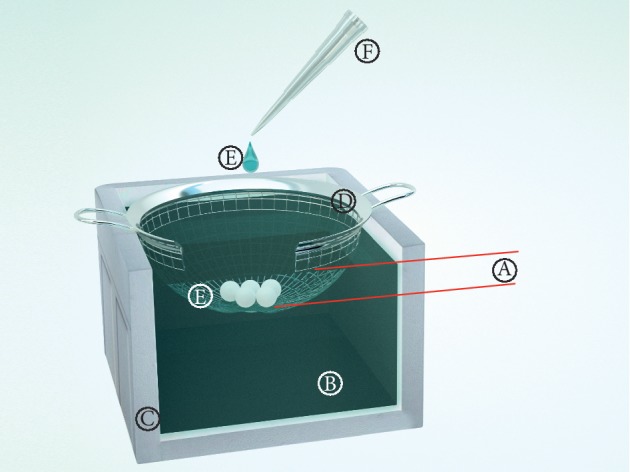
Scheme of spermatozoa vitrification. A, distance between the bottom of the strainer and the surface of the liquid agent (minimum 3 cm); B, liquid agent (liquid nitrogen or liquid air); C, foam box; D, strainer; E, spermatozoa suspension in the form of balls; F, pipette for the formation of 20 *μ*l granules.

Spermatozoa after DGC (as described above) were centrifuged at 300 g for 10 min. The pellets were resuspended in vitrification medium to achieve a concentration of 15 × 10^6^ spermatozoa/ml, with subsequent incubation for 5 min at 37°C in 5% CO_2_. Cooling in the liquid agent (nitrogen or air) was carried out as illustrated in Figure 2 and described in [4]. In brief, using a micropipettor (Eppendorf, Hamburg, Germany) at a distance of 10 cm from respective liquid agent, 20 *µ*l aliquots of spermatozoa suspension were dropped directly into this cooling agent. A sphere immediately forms and floats to the surface. After 6 s in liquid nitrogen and 8 s in liquid air, the sphere solidifies and falls to the bottom of the strainer. After solidification, the spheres were collected, packaged into 1.8 ml cryotubes, and stored for at least 24 h in a tank with liquid nitrogen before warming. Warming was performed by quickly submerging spheres, one-by-one (not more than five spheres), into 5 ml HTF with 1% DSS prewarmed to 37°C, accompanied by gentle vortexing for 10 s. The warmed sperm suspension was maintained at 37°C in 5% CO_2_ for 10 min and then centrifuged at 380 g for 5 min. The cell pellet was finally resuspended in 50 *µ*l HTF with 1% DSS for the evaluation of quality.

### 2.4. Spermatozoa Motility

Motility of spermatozoa was assessed immediately after ejaculation and after each spermatozoa preparation technique (DGC) and after warming using Makler's counting chamber (0.01 mm^2^ and 10 *μ*m deep) to calculate. Motility was estimated under the light microscope (Zeiss, Goettingen, Germany) at 400x magnification [4]. Only spermatozoa with progressive motility, categories “a” (rapid and regular forward progression) and “b” (moderate, slow, or sluggish forward progression) according to the WHO guidelines [29] were assessed. The percentage of progressively (*a* + *b*) motile spermatozoa was determined according to the following equation: (*a* + *b* motile spermatozoa/total spermatozoa) × 100.

### 2.5. Viability by SYBR-14 and Propidium Iodide

The samples were processed using SYBR-14 and propidium iodide (PI), using the dead/live spermatozoa viability staining kit (L-7011, Molecular Probes Inc., Eugene, USA) according to the method described in [31]. Spermatozoa suspension (45 *μ*l, 25–50 × 10^6^/ml) was placed in an Eppendorf tube and 5 *μ*l each of SYBR-14 in DMSO (1 *μ*M) and PI (120 *μ*M) in BSA-saline (1 mg/ml) was added and incubated at 37°C for 10 min. Three 10 *μ*l drops of the mix were placed on a slide under coverslips and evaluated under epifluorescence using Nikon B-2A filter block at 400x magnification. Researchers can distinguish live and dead cells with visible-light excitation, avoiding the harmful effects of UV exposure. When spermatozoa was incubated briefly with these two stains, live spermatozoa cells with intact cell membranes fluoresce bright green, while cells with damaged cell membranes fluoresce red. Both live (green) and dead (red) spermatozoa are seen simultaneously. A total of 200 spermatozoa per sample were assessed. SYBR-14 is a membrane-permeant and nonfluorescent compound, which is immediately deacylated and converted into high fluorescent compounds by intracellular esterases. These green fluorochromes are maintained intracellular by intact membranes. As plasma membranes deteriorate at cell death, cells lose their ability to resist the influx of red fluorescent PI which replaces or quenches green fluorochromes.

### 2.6. FITC Annexin V with 7-AAD Apoptosis Assay

To perform this assay, we used the FITC Annexin V Apoptosis Detection Kit with 7-AAD (Biolegend, San Diego, USA). Spermatozoa cells were washed twice with cold cell staining buffer and then resuspended in Annexin V Binding Buffer at a concentration of 0.25–1.0 × 10^6^ cells/ml. After that, 100 *μ*l of cell suspension was transferred in a 5 ml test tube. Spermatozoa cells were labelled with 5 *μ*l of FITC Annexin V and 7-AAD viability staining solution. The cells were gently vortexed and incubated for 15 min at room temperature (25°C) in the dark. One drop of Dabco and 10 *μ*l of stained spermatozoa sample were placed on a slide, gently mixed, then covered with a coverslip, and sealed with nail varnish. Slides were observed using appropriate filters. A total of 200 spermatozoa per sample were assessed.

Early apoptotic cells will exclude 7-AAD (7-amino-actinomycin D), while late-stage apoptotic cells will stain positively, due to the passage of these dyes into the nucleus where they bind to DNA. 7-AAD has a high DNA-binding constant and is efficiently excluded by intact cells. When excited by 488 laser light, 7-AAD fluorescence is detected in the far red range of the spectrum (650 nm long-pass filter).

Annexin V is a member of the annexin family of intracellular proteins that binds to phosphatidylserine (PS) in a calcium-dependent manner. PS is normally only found on the intracellular leaflet of the plasma membrane in healthy cells, but during early apoptosis, membrane asymmetry is lost and PS translocates to the external leaflet. Fluorochrome-labelled Annexin V can then be used to specifically target and identify apoptotic cells. To help distinguish between the necrotic and apoptotic cells we use the 7-AAD solution.

### 2.7. Statistical Analysis

For the statistical analysis, we used one-way nonparametric analysis of variance (ANOVA) with a significance level of 0.05. The Bonferroni multiple comparisons test was used to establish differences between the groups. Results are represented as mean ± SEM throughout the study. *p* < 0.05 was considered statistically significant. All analyses were done using SPSS version 17 for Windows (SPSS, Chicago, IL, USA) and performed with the Prism 6 software (GraphPad, La Jolla, USA).

## 3. Results

**Figure 3:**
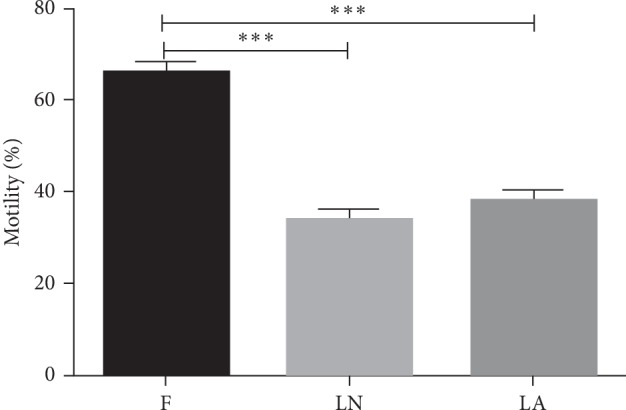
Progressive motility of vitrified spermatozoa. F, fresh spermatozoa (control group); LN, group of spermatozoa vitrified in liquid nitrogen; LA, group of spermatozoa vitrified in liquid air. Data are expressed as mean ± SEM. Significant difference versus control, ^*∗∗∗*^*p* < 0.001.

The process of vitrification and warming markedly affected the motility of the spermatozoa (Figure 3). While the fresh control spermatozoa showed 65.9 ± 2.5% of progressive motility, this rate was reduced in the LN and LA groups (to 34.0 ± 1.9% and 38.1 ± 2.3%, respectively) (*p* < 0.001 compared to the control). It was detected no difference in the motility rates of the two treatment groups (LN and LA) (*p* > 0.1).

**Figure 4:**
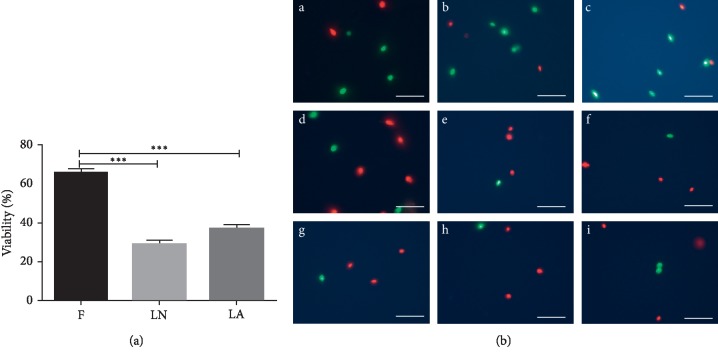
Viability of vitrified spermatozoa. F, fresh spermatozoa (control group); LN, group of spermatozoa vitrified in liquid nitrogen; LA, group of spermatozoa vitrified in liquid air. Micrographs of fresh and treated spermatozoa (right figure). (A (a, b, c)) fresh spermatozoa (control group), (B (d, e, f)) spermatozoa vitrified in liquid nitrogen, and (C (g, h, i)) spermatozoa vitrified in liquid air. (D) A total of 200 spermatozoa were assessed, and the results are shown on the right. ^*∗∗∗*^Significant difference versus control, *p* < 0.001. Data are expressed as mean ± SEM, Scale bars: 50 *μ*m.

The influence of the two vitrification methods is shown in Figure 4. Viable spermatozoa cells with intact cell membranes are bright green, while cells with damaged cell membranes are red. The viability of spermatozoa decreased significantly after cryopreservation: from 65.6 ± 2.2% in the control (“fresh”) group to 29.0 ± 1.8% and 36.6 ± 2.6% in the LN and LA groups, respectively (both *p* < 0.001 vs. the control). However, no significant differences were observed when comparing the LN and LA groups (*p* > 0.05) (Figure 4).

**Figure 5:**
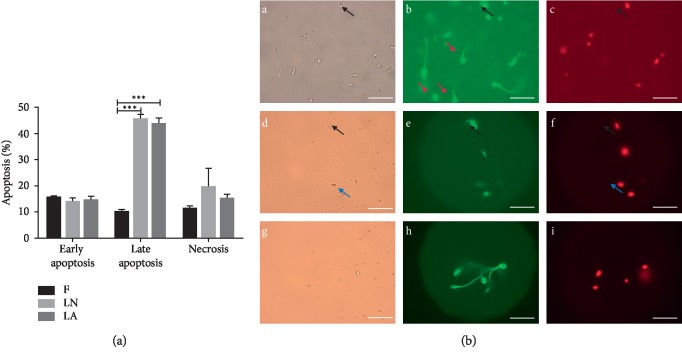
Apoptosis of vitrified spermatozoa. F, fresh spermatozoa (control group); LN, group of spermatozoa vitrified in liquid nitrogen; LA, group of spermatozoa vitrified in liquid air. Micrographs of fresh and treated spermatozoa (right figure). (A (a, b, c)) fresh spermatozoa (control group), (B (d, e, f)) spermatozoa vitrified in liquid nitrogen, and (C (g, h, i)) spermatozoa vitrified in liquid air. (D) A total of 200 spermatozoa were assessed, and the results are shown on the right. ^*∗∗∗*^Significant difference versus control, *p* < 0.001. Data are expressed as mean ± SEM. Scale bars: 50 *μ*m.

Apoptosis (early apoptosis, late apoptosis, and necrosis) data are shown in Figure 5. When comparing the control (“fresh”) and treatment groups, the rates of early apoptosis were not significantly different (15.4 ± 0.8%, 13.8 ± 1.5%, and 14.3 ± 1.8% for Groups F, LN, and LA, respectively) (*p* > 0.1). It was detected a greater proportion of spermatozoa in late apoptosis in the LN (45.5 ± 1.8%) and LA (43.7 ± 2.2%) groups than in fresh samples (10.0 ± 1.1%) (*p* < 0.001) (Figure 5). The late apoptosis rates of spermatozoa in LN and LA groups were similar (*p* > 0.1).

The rate of necrosis increased from 11.2 ± 1.1% in the control group to 19.5 ± 2.0% for the LN group (*p* < 0.05). The increase in the rate of necrosis was not significant in Group LA (15.0 ± 1.8%, *p* > 0.1). We detected no significant difference in the necrosis rates when comparing the LN and LA groups (*p* > 0.05).

## 4. Discussion

The data regarding the risk of cross contamination during cryopreservation of reproductive cells in liquid nitrogen are brightly presented [22–26, 32–36]. It was shown that viruses, bacteria, and mycoplasmas survive after cooling in liquid nitrogen.

It has been established that viruses have especially high cryostability [21, 36–38].

Bacteria also have high cryostability [39], as demonstrated by the majority of recent publications regarding freezing-drying (cryoprotectant-free freezing with the following vacuumization) of lactic acid bacteria [40].

Mycoplasma are very cryostable pathogens. For example, *Mycoplasma equigenitalium* and *Mycoplasmasubdolum* show high viability rates after cryoprotectant-free cryopreservation by direct plunging into liquid nitrogen and storage at −196°C for 30 days [41]. Mycoplasma can also be effectively freeze-dried [42].

Recently, a new device to produce aseptic liquid air has been developed and successfully used in the vitrification of human oocytes and seen as a breakthrough for safe vitrification using open systems [27]. The vitrification of human pronuclear oocytes through direct contact of oocytes with a clean cooling agent has been reported [28]. However, there are limited data about the vitrification of human spermatozoa through direct contact with clean liquid air. Therefore, the aim of our investigations was to test the new method of cryoprotectant-free vitrification (directly into liquid nitrogen and aseptic air) on human spermatozoa motility, viability, and apoptosis.

Previously, it was reported a positive effect of sterile liquid air by vitrification of human oocytes and embryos [27, 28]. By our supposition, the use of clean liquid air can be beneficial for cryopreservation spermatozoa by direct plunging into liquid air. The results in our study showed that there was no difference found between parameters of viability of spermatozoa vitrified in liquid nitrogen (with risk of microbial contamination) and spermatozoa vitrified in clean liquid air. We have established that cooling in clean liquid air was also effective but without the risk of contamination.

In Arav et al.'s study [27], the vitrification and storage experiments on mice embryos and human oocytes were performed. Results showed similar cooling rates by plunging of cells into liquid nitrogen and liquid air. Bioburden tests of CLAir and Esther showed no contamination of cells, while massive contamination was found in “commercial” liquid nitrogen and storage canisters. Mice blastocysts had a survival rate of over 90%, with 80% hatching rate after vitrification in CLAir and 1 week storage in Esther, similar to the fresh (control) results. Human oocytes vitrified in CLAir and in liquid nitrogen for three consecutive vitrification/warming cycles showed high survival rate reflected to reexpansion of embryos in both groups. These new systems represent a breakthrough for safe vitrification using open systems and a safe storage process generally [27].

Arav et al. [27] also showed that clean liquid air has the similar cryogenic properties as liquid nitrogen. The authors noted that liquefied clean liquid air has practically the same temperature to that of liquid nitrogen and is able to guarantee the similar cooling rate. The rates “progressive motility,” “viability,” “late apoptosis,” and “necrosis” for spermatozoa in LN and LA groups were similar. However there was a statistical tendency for increasing rates of “progressive motility” and “viability” and decreasing rates of “apoptosis” and “necrosis” when comparing spermatozoa vitrified in liquid air vs. liquid nitrogen.

For described experiments, it was used FITC Annexin V with 7-AAD kit to detect apoptosis. FITC Annexin V staining precedes the loss of membrane integrity which accompanies the latest stages of cell death resulting from either apoptotic or necrotic processes. Apoptosis staining identified the different subpopulations of spermatozoa: (a) early apoptotic spermatozoa with exteriorized phosphatidylserine (PS) receptor and intact plasmalemma (Annexin V^+^/7-AAD^−^), (b) late apoptotic spermatozoa with PS receptor translocation and leaky plasmalemma (Annexin V^+^/7-AAD^+^), and (c) dead spermatozoa with damaged plasmalemma with no detectable PS receptor (Annexin V^−^/7-AAD^+^) [43–45].

## 5. Conclusion

Cryoprotectant-free vitrification by the direct dropping of human spermatozoa into clean cooling agent (liquid air) is a good alternative to the use of nonsterile liquid nitrogen, thereby minimising the risk of microbial contamination.

## Data Availability

The data used to support the findings of this study are included within the article.

## References

[1] Sanger W. G., Olson J. H., Sherman J. K. (1992). Semen cryobanking for men with cancer-criteria change. *Fertility and Sterility*.

[2] Jensen J. R., Morbeck D. E., Coddington C. C. (2011). Fertility preservation. *Mayo Clinic Proceedings*.

[3] Polge E. J. C. (1957). Low-temperature storage of mammalian spermatozoa. *Proceedings of the Royal Society of London Series B-Biological Sciences*.

[4] Isachenko E., Isachenko V., Weiss J. M. (2008). Acrosomal status and mitochondrial activity of human spermatozoa vitrified with sucrose. *Reproduction*.

[5] Di Santo M., Tarozzi N., Nadalini M., Borini A. (2012). Hduman sperm cryopreservation: update on techniques, effect on DNA integrity, and implications for ART. *Advances in Urology*.

[6] Pabón D., Meseguer M., Sevillano G. (2019). A new system of sperm cryopreservation: evaluation of survival, motility, DNA oxidation, and mitochondrial activity. *Andrology*.

[7] Said T. M., Gaglani A., Agarwal A. (2010). Implication of apoptosis in sperm cryoinjury. *Reproductive Biomedicine Online*.

[8] Fraga C. G., Motchnik P. A., Shigenaga M. K., Helbock H. J., Jacob R. A., Ames B. N. (1991). Ascorbic acid protects against endogenous oxidative DNA damage in human sperm. *Proceedings of the National Academy of Sciences*.

[9] Hammerstedt R. H., Graham J. K. (1992). Cryopreservation of poultry sperm: the enigma of glycerol. *Cryobiology*.

[10] Mossad H., Morshedi M., Toner J. P., Oehninger S. (1994). Impact of cryopreservation on spermatozoa from infertile men: implications for artificial insemination. *Archives of Andrology*.

[11] Petrunkina A. M. (2007). Fundamental aspects of gamete cryobiology. *Journal of Reproductive Medicine and Endocrinology*.

[12] Gilmore J. A., Liu J., Gao D. Y., Critser J. K. (1997). Determination of optimal cryoprotectants and procedures for their addition and removal from human spermatozoa. *Human Reproduction*.

[13] Isachenko V., Maettner R., Petrunkina A. M. (2012). Vitrification of human ICSI/IVF spermatozoa without cryoprotectants: new capillary technology. *Journal of Andrology*.

[14] Chen Y., Li L., Qian Y. (2015). Small-volume vitrification for human spermatozoa in the absence of cryoprotectants by using Cryotop. *Andrologia*.

[15] Nawroth F., Isachenko V., Dessole S. (2002). Vitrification of human spermatozoa without cryoprotectants. *CryoLetters*.

[16] Isachenko V., Isachenko I. I., Katkov S., Dessole, Nawroth F. (2003). Vitrification of mammalian spermatozoa in the absence of cryoprotectants: from past practical difficulties to present success. *Reproductive Biomedicine Online*.

[17] Isachenko V., Isachenko E., Montag M. (2005). Clean technique for cryoprotectant-free vitrification of human spermatozoa. *Reproductive BioMedicine Online*.

[18] Zhu J., Jin R.-T., Wu L.-M. (2014). Cryoprotectant-free ultra-rapid freezing of human spermatozoa in cryogenic vials. *Andrologia*.

[19] Sánchez R., Risopatrón J., Schulz M., Villegas J. V., Isachenko V., Isachenko E. (2012). Vitrified sperm banks: the new aseptic technique for human spermatozoa allows cryopreservation at −86°C. *Andrologia*.

[20] Agha-Rahimi A., Khalili M. A., Nottola S. A., Miglietta S., Moradi A. (2016). Cryoprotectant-free vitrification of human spermatozoa in new artificial seminal fluid. *Andrology*.

[21] Isachenko V., Rahimi G., Mallmann P., Sanchez R., Isachenko E. (2017). Technologies of cryoprotectant-free vitrification of human spermatozoa:asepticity as criterion of effectiveness. *Andrology*.

[22] Bielanski A. (2005). Non-transmission of bacterial and viral microbes to embryos and semen stored in the vapour phase of liquid nitrogen in dry shippers. *Cryobiology*.

[23] Bielanski A. (2005). Experimental microbial contamination and disinfection of dry (vapour) shipper Dewars designed for short-term storage and transportation of cryopreserved germplasm and other biological specimens. *Theriogenology*.

[24] Bielanski A., Bergeron H., Lau P. C. K., Devenish J. (2003). Microbial contamination of embryos and semen during long term banking in liquid nitrogen. *Cryobiology*.

[25] Bielanski A., Lalonde A. (2009). Effect of cryopreservation by slow cooling and vitrification on viral contamination of IVF embryos experimentally exposed to bovine viral diarrhea virus and bovine herpesvirus-1. *Theriogenology*.

[26] Bielanski A. (2012). A review of the risk of contamination of semen and embryos during cryopreservation and measures to limit cross-contamination during banking to prevent disease transmission in ET practices. *Theriogenology*.

[27] Arav A., Natan Y., Levi-Setti P. E., Menduni F., Patrizio P. (2016). New methods for cooling and storing oocytes and embryos in a clean environment of −196°C. *Reproductive Biomedicine Online*.

[28] Isachenko V., Todorov P., Seisenbayeva A. (2018). Vitrification of human pronuclear oocytes by direct plunging into cooling agent: non sterile liquid nitrogen vs. sterile liquid air. *Cryobiology*.

[29] World Health Organization (2010). *WHO Laboratory Manual for the Examination and Processing of Human Semen*.

[30] Quinn P., Kerin J. F., Warnes G. M. (1985). Improved pregnancy rate in human in vitro fertilization with the use of a medium based on the composition of human tubal fluid∗∗supported in part by a grant from the National Health and Medical Research Council of Australia. *Fertility and Sterility*.

[31] Garner D. L., Johnson L. A. (1995). Viability assessment of mammalian sperm using SYBR-14 and propidium Iodide1. *Biology of Reproduction*.

[32] Benson E. E. (2008). Cryopreservation of phytodiversity: a critical appraisal of theory & practice. *Critical Reviews in Plant Sciences*.

[33] Mortimer D. (2004). Current and future concepts and practices in human sperm cryobanking. *Reproductive BioMedicine Online*.

[34] Pomeroy K. O., Harris S., Conaghan J. (2010). Storage of cryopreserved reproductive tissues: evidence that cross-contamination of infectious agents is a negligible risk. *Fertility and Sterility*.

[35] Rall W. F. (2003). Avoidance of microbial cross-contamination of cryopreserved gametes, embryos, cells and tissues during storage in liquid nitrogen. *Embryology Newsletter*.

[36] Tomlinson M. (2008). Risk management in cryopreservation associated with assisted reproduction. *Cryo Letters*.

[37] Hansen L. J. J., Daoussi R., Vervaet C., Remon J.-P., De Beer T. R. M. (2015). Freeze-drying of live virus vaccines: a review. *Vaccine*.

[38] Stanger J., Wong J., Conceicao J., Yovich J. (2012). Vitrification of human embryos previously cryostored by either slow freezing or vitrification results in high pregnancy rates. *Reproductive Biomedicine Online*.

[39] Stringfellow D. A., Wolfe D. F., McGuire J. A., Lauerman L. H., Gray B. W., Sparling P. H. (1986). Effects of embryo-freezing and thawing techniques on the survivability of. *Theriogenology*.

[40] Polo L., Mañes-Lázaro R., Olmeda I. (2017). Influence of freezing temperatures prior to freeze-drying on viability of yeasts and lactic acid bacteria isolated from wine. *Journal of Applied Microbiology*.

[41] Bermudez V., Miller R., Johnson W., Rosendal S., Ruhnke L. (1988). Effect of sample freezing on the isolation of Mycoplasma spp. from the clitoral fossa of the mare. *Canadian Journal of Veterinary Research*.

[42] Yugi H., Suzuki M., Sato S., Ozaki Y. (1973). Freeze-drying of mycoplasma. *Cryobiology*.

[43] Wechalekar H., Setchell B. P., Breed W. G., Ricci M., Leigh C., Peirce E. (2009). 115. Whole body heat exposure induces apoptosis in mouse caudal epididymal spermatozoa. *Reproduction, Fertility and Development*.

[44] Sawicka D., Chojnacka-Puchta L., Zielinski M., Plucienniczak G., Plucienniczak A., Bednarczyk M. (2015). Flow cytometric analysis of apoptosis in cryoconserved chicken primordial germ cells. *Cellular and Molecular Biology Letters*.

[45] Allam J.-P., Fronhoffs F., Fathy A. (2008). High percentage of apoptotic spermatozoa in ejaculates from men with chronic genital tract inflammation. *Andrologia*.

